# Synthesis, Development and Characterization of Nanotubes

**DOI:** 10.3390/nano13111762

**Published:** 2023-05-30

**Authors:** Marius Dobromir

**Affiliations:** Department of Exact and Natural Sciences, Institute of Interdisciplinary Research, Alexandru Ioan Cuza University of Iasi, Blvd. Carol I, No. 11, 700506 Iasi, Romania; marius.dobromir@uaic.ro

In recent decades, the great demand for device miniaturization has attracted the attention of researchers focused on the growth, modification, properties, and applications of one-dimensional nanostructures, such as nanotubes. Nanotubes are cylindrical structures with diameters of 1–100 nm. In recent years, nanotubes synthesized from different types of materials (i.e., carbon, CNTs; titanium, NTAs; and tellurium, TeNTs) have been selected as promising alternative materials for various applications based on their characteristic properties. CNTs are electrically insulating, semiconducting, or exhibit metallic conductance; NTAs have a large area/volume ratio and fast electron transport, as well as a low recombination rate of charge carriers, which enable increased photocatalytic efficiency and durability; and TeNTs exhibit a tunable bandgap, high carrier mobility, and high thermal conductivity. The variety of applications covered by the eight articles published in the Special Issue is proof of the growing attention paid to the use of nanotube materials for electronic devices, photocatalytic devices, environmental electronics devices, biosensors, etc.

In this Special Issue, the research articles are focused on the following:

Carbon nanotubes: when combined with Pd nanoparticles loaded onto Co_3_O_4_, they serve as a promising cathode catalyst for enhancing the electrocatalytic activity and oxygen reduction reaction at the cathode in direct urea fuel cells [1]; they decrease the incidence and severity of *A. solani*, and increase the fruit yield of tomato crop and dry shoot biomass [2]; they are used as electromechanical sensing devices when doped with resins [3]; they are used for the non-covalent functionalization and dispersion of two different S-layer proteins for the development of novel materials, such as biosensors [4]; when coupled with reduced graphene oxide (rGO) on a Si substrate, they lead to the formation of electrically conductive nanostructures for interconnections in nanoelectronics [5]; when properly dispersed in a liquid crystal matrix, they present dielectric properties considerably different from those of the pure liquid crystal [6].

Titania nanotubes: the defect creation within the tube walls and the changes in surface morphology after ion bombardment constitute a versatile tool to achieve well-defined and tunable topographies and distinct surface characteristics [7].

Tellurium nanotubes: due to their tunable bandgap, high carrier mobility, high thermal conductivity, and in-plane anisotropy, they are proven to be versatile and applicable in sensing and decontamination, energy storage, catalysts, and can form heterostructures with other nanomaterials [8].

The obtained results are expected to be useful for researchers working in the field of nanotubes.
